# Equal school meals for all - differentiation of school meals according to school social index

**DOI:** 10.1016/j.puhip.2025.100693

**Published:** 2025-12-11

**Authors:** Cordula Hundeshagen, Heike Rosmann

**Affiliations:** Food and Consumer Education, IES Landau, Institute of Environmental Sciences, RPTU Kaiserslautern-Landau, Fortstraße 7, 76829, Landau, Germany

**Keywords:** School food standard, Nutritious school food, School social index, School food policy, Nutrition inequality

## Abstract

**Objectives:**

The EU is discussing providing all children healthy food at least once a day through school lunch. In Germany, a School Food Standard (SFS) aims to ensure healthy and sustainable meals, however its implementation is mandatory in only some federal states, while voluntary in others. To address social disadvantages, some states use school social indices to allocate additional funding to disadvantaged schools. This study analyzes German school menus to assess their compliance with SFS requirements under both voluntary and mandatory implementation and examines whether healthy, sustainable lunches are accessible regardless of schools' socioeconomic status.

**Study design:**

Exploratory school meal analysis.

**Methods:**

A random sample of schools was selected based on the school register and social index, and schools were invited to participate voluntarily. Menus from a state with obligatory (OSFS) and voluntary (VSFS) implementation of the SFS were collected and analyzed. The analysis focused on SFS criteria, including a mixed diet menu, ovo-lacto-vegetarian dishes and criteria for menu planning. Binomial logistic regression and two-step multiple regression models were used to assess the influence of the school social index on compliance with SFS requirements.

**Results:**

We analyzed 67 OSFS menus and 79 VSFS menus. No significant influence of the school social index on menu quality was observed. OSFS menus generally met SFS requirements to a greater extent and exhibited a healthier dietary pattern. OSFS menus included more grain products, vegetables, legumes, and salad, fruits, and fatty fish, while containing fewer potato products, fried items, and industrial meat substitutes. They also used more organic products and sustainably sourced fish and provided better labeling for animal species, meat substitutes, additives, and allergens. OSFS menus featured more ovo-lacto-vegetarian options and prioritized seasonal and regional products. However, OSFS menus were less tailored to target audiences, lacked nutritional information, and often failed to clearly describe meal components.

**Conclusion:**

Regardless of socioeconomic status, all students receive the same food. OSFS menus better fulfilled SFS requirements and are a healthier and more sustainable option. However, compliance remains incomplete, even in OSFS states. Further research is needed to address implementation barriers and ensure equitable access to healthy school meals.

## 1 Introduction

In 2024, 15.5 % of Germany's population experienced monetary poverty, and 20.9 % were affected by poverty and social exclusion [1]. Around 3.2 million people face food insecurity [2], including many children [3].

Research indicates that children and adolescents from lower socio-economic status (SES) consume fewer fruits and vegetables compared to their peers from higher SES, while exhibiting a higher intake of sugar-sweetened beverages, meat and meat products, as well as fast food [[4], [5], [6], [7]]. This issue also affects children and adolescents, making them particularly vulnerable to the associated health risks [[8], [9], [10]]. The German Health Interview and Examination Survey for Children and Adolescents (KiGGS) shows that 15 % of children aged 3 to 17 are overweight and 6 % are obese. Overweight and obesity are significantly more common among children from lower socioeconomic backgrounds compared to those from higher SES families [11].

A strategy is needed to address the link between poverty and poor dietary habits. School meals are an effective intervention for promoting healthier dietary habits, with empirical evidence highlighting their role in improving children's health, well-being, and educational performance [12,13]. In Finland, Raulio et al. found that schoolchildren who regularly consumed school meals were more likely to make food choices aligned with nutritional recommendations compared to those who used school catering services less frequently [14]. Holford and Rabe's analysis of the impact of free school meals on the body weight of participating students revealed a decline in the prevalence of obesity and an increase in the prevalence of healthy body weight [15].

Effective school feeding programs rely on key components, particularly the provision of nutritionally balanced meals, which are supported through the implementation of school food standards (SFS). All EU member states, along with Norway, the United Kingdom, and Switzerland, have established SFS to prevent obesity and promote health [16]. About half of these countries enforce legally binding regulations, while the others rely on voluntary adherence [16].

In Germany, the German Nutrition Society (DGE) introduced a quality standard for school feeding (DGE-Qualitätsstandard Schule) in 2007 [17]. However, as education policy is regulated at the state level, implementing these standards remains voluntary in most states.

Studies show that obligatory school food standards (OSFS) lead to improved meal quality [[18], [19], [20], [21], [22], [23]]. For example, the updated SFS introduced in the United States in 2012 led to reduced consumption of solid fats and added sugars [24], increased availability of whole grains, vegetables, greens, and beans, and decreased refined grains and sodium in school menus [25]. This was accompanied by a decline in overweight and obesity among children consuming SFS-compliant school meals [24,26].

Similar benefits were observed in Canada [27] and primary schools in North East England [28].

In Germany, our study on voluntary and OSFS implementation found that school meals in states with OSFS are more aligned with nutritional recommendations and include more healthy and sustainable foods [29]. Still, the debate continues over whether mandatory or voluntary programs are more effective in ensuring healthy meals for children from all backgrounds.

Healthier menu options are more common in wealthier areas [30], while economically disadvantaged areas often have more fast-food options near schools [31]. Using the German Index of Socioeconomic Deprivation (GISD) of the Robert Koch Institute, our research found no clear link between socioeconomic status and school meal quality under mandatory or voluntary standards [29].

The GISD reflects regional indicators like education, income, and occupation at the district and municipality levels [32]. But it does not capture differences within city districts or individual schools. To support schools in disadvantaged areas and improve learning environments, some federal states offer targeted funding based on school social indices that assess school-level socioeconomic status [33].

It remains uncertain whether students from all socioeconomic backgrounds are offered healthy school lunches. Therefore, the aim of this study is to analyze German school menus in terms of their compliance with SFS requirements under voluntary and mandatory implementation, and to determine whether healthy and sustainable lunches are accessible to schools regardless of their socioeconomic context.

## 2 Methods

### 2.1 Study design and sampling method

Social indices are used to assess disparities between different social school environments and serve as educational policy tools to promote educational equity. They help identify schools with predominantly socially and economically disadvantaged students, allowing for targeted, needs-based resource allocation. The aim is to highlight social diversity and ensure fairer distribution of educational resources [34].

At the time of the study, only five German federal states systematically used school social indices management [35]. For this study, meal plans from North Rhine-Westphalia (with VSFS) and Hamburg (with OSFS) were analyzed due to their comparable index structures.

In North Rhine-Westphalia, the social index is calculated using four indicators: (1) the share of children living in poverty, (2) the proportion of students from non-German-speaking households, (3) those who migrated from abroad, and (4) students with special educational needs. A confirmatory factor analysis determines the index values, categorizing schools into nine levels, with higher levels indicating greater support needs [36].

In Hamburg, the social index was calculated using confirmatory factor analysis based on eight variables: (1) the proportion of students with a non-German family language, (2) students with special educational needs, (3) the proportion of students receiving benefits under a special education and participation package, (4) the upper secondary school graduation rate, (5) share of voter turnout in the area, (6) the proportion of individuals receiving educational financial assistance, (7) unemployment rates, and (8) the proportion of children receiving minimum income support. The index ranges from one to six, with lower values indicating higher support needs [37].

As part of an exploratory assessment of the composition of school food menus in the selected federal states we combined the school registries of general schools with the social indices and drew a convenience sample of 30 schools per federal state by each social index category using the random sampling function in Excel (Version, Microsoft, 2016). Schools were contacted by email in autumn 2023 to provide four-week lunch menu cycles with 20 supply days. A follow-up email was sent after three weeks, followed by a telephone follow-up to improve the response rate. If the school food caterer was identified but menus were not available directly from the school, the caterers were contacted directly to obtain the menus. In Hamburg, it was often possible to identify the caterer through the school's website and download the menu directly from the caterer's website.

Lunch menus were stratified based on whether they originated from North-Rhine Westphalia as a federal state with voluntary implementation of SFS criteria or Hamburg as obligatory implementation state.

### 2.2 DGE school food standard

The analysis of school menus was based on the 2023 quality standards of the German Nutrition Society (DGE SFS), which include six quality areas: the development of quality school meals, the design of health-promoting and sustainable meals with specified food qualities and frequencies for both mixed and ovo-lacto-vegetarian diets, additional criteria for menu planning, criteria for the use of convenience food in mass catering, menu-specific criteria, and considerations for the living environment [38]. Only requirements that could be directly assessed from the menus were included in the analysis, excluding aspects like low-fat preparation, reduced use of sugar and salt, and the design of break rooms were excluded.

Supplementary Table 1 summarizes all requirements and indicates which ones were included in the analyses. Table 1 presents an overview of the analyzed requirements, organized by quality group, along with their corresponding coding guidelines.

**Table 1:** Analyzed SFS requirements and their coding guidelines.

Requirements	Coding guidelines

**Design of the meal food qualities and frequencies for lunch, mixed diet, 4 weeks with 20 catering days**	

5x (1x daily) grain/-products/potatoes	Yes: if grain, grain products or potatoes have been used once a day in 20 days, otherwise No
of which at least 1x whole grain products	Yes: if at least once a week whole grain products have been used, No: if not fulfilled, or students get no whole grain by selecting an offered alternative menu
of which max. 1x potato products	Yes: if at most once a week potato products have been used, No: if more potato products have been used, or students get more potato products by selecting an offered alternative menu
5x (1x daily) vegetables, legumes or salad	Yes: if vegetables, legumes or salad have been used once a day in 20 days, otherwise No
of which at least 2x raw vegetables or salad	Yes: if at least twice a week raw vegetables or salad have been used, otherwise No
of which at least 1x legumes	Yes: if at least once a week legumes have been used, otherwise No
At least 2x fruits	Yes: if at least twice a week fruits have been used, otherwise No
of which at least 1x whole fruits	Yes: if at least once a week whole fruits have been used, otherwise No
At least 2x milk and milk products	Yes: if at least twice a week milk and milk products have been used, otherwise No
Max 1x meat and sausages	Yes: if at most once a week meat and sausages have been used, No: if more meat and sausages were offered or students get more meat and sausages by selecting an offered alternative menu (not the SFS menu)
of which at least 2x lean meat	Yes: if it was at least two times lean meat in 20 catering days, No: if no meat type was mentioned or not two times lean meat was offered All white meat was considered as lean meat
At least 1x sea fish	Yes: if it was at least once a week fish has been used, otherwise No
of which at least 2x fatty sea fish	Yes: if it was at least twice a month fatty sea fish was used, No: not twice a week fatty fish was offered or no fish type was mentioned Fish over 10 % fat, like Salmon, mackerel, tuna, herring, sardines, trout, anchovies and sturgeon were treated as fatty fish.

**Food qualities and frequencies for a lunch, ovo-lacto-vegetarian diet,** 4 weeks with 20 catering days	

5x (1x daily) grain/-products/potatoes	Yes: if grain, grain products or potatoes have been used once a day in 20 days, otherwise No
of which at least 1x whole grain products	Yes: if at least once a week whole grain products have been used, otherwise No
of which max. 1x potato products	Yes: if at most once a week potato products have been used, otherwise No All potato products that were not offered in their original form were regarded as potato products.
5x (1x daily) vegetables, legumes or salad	Yes: if vegetables, legumes or salad have been used once a day in 20 days, otherwise No
of which at least 2x raw vegetables or salad	Yes: if at least twice a week raw vegetables or salad have been used, otherwise No
of which at least 1x legumes	Yes: if at least once a week legumes have been used, otherwise No
At least 2x fruits, nuts and oil seeds	Yes: if at least twice a week fruits have been used, otherwise No
of which at least 1x whole fruits	Yes: if at least once a week whole fruits have been used, otherwise No
Of which at least 1x nuts and oil seeds	Yes: if at least once a week nuts and oil seeds have been used, otherwise No
At least 2x milk and milk products	Yes: if at least twice a week milk and milk products have been used, otherwise No



**Additional criteria for menu planning**	

Ovo-lacto-vegetarian dish available on a daily basis	Yes, if every day a ovo-lacto-vegetarian dish was offered, otherwise No
Seasonal offer preferred (seasonal fruits and vegetables)	Yes, if at least one type of fruit or vegetable from the seasonal range has been identified, otherwise No
Regional food products are used	Yes, if fruits and vegetables which can be grown in Europe were offered, No: if non-European fruits and vegetables were offered or no kind of fruits and vegetables are mentioned
Grain, grain products and potatoes are varied provided	Yes, a variance in the offer was guaranteed and the same component were not offered for several days in a row.
Max. 4x fried/breaded products in 20 days	Yes, if at most once a week fried/breaded products are used, otherwise No
Max. 4x industrial produced meat substitutes in 20 days	Yes, if at most once a week industrial produced meat substitutes are used, otherwise No
Menu cycle is at least four weeks	Yes, if the menu cycle repeats at least after four weeks, otherwise No
The dishes are varied	Yes, Yes, if the menus vary and are visually appealing. If, on multiple occasions over five days, there was only one carbohydrate option with a vegetable-free sauce, the criterion was rated as No.

**Menu criteria**	

Additives declared	Yes, if declared, otherwise No
Allergens declared	Yes, if declared, otherwise No
Nutrition labelling in compliance with legal requirements	Yes, if under legal compliance, N/A no nutritional values declared No, if they were not declared correctly
Nutrient optimized menus emphasized	Yes, if the nutrient-optimized menu (DGE-menu)) was highlighted by placement in the first position, colors, symbols or naming, No: if no nutrient optimized menu was offered or if it was not emphasized
Unambiguous designation of meals or further explanation	Yes, if all components of a dish were clearly recognizable or described in more detail
For meat, sausages and fish the animal species are named	Yes, if the species are named, otherwise No
The basis of animal substitute products (meat, fish, egg, milk, milk-products) is clearly labelled	Yes: if the basis of the animal substitute is always mentioned, otherwise No
If prices are shown, they are clearly displayed	Yes, if they are clearly displayed, e.g. by potion or 100 g, N/A, if no prices are mentioned, No, if they were not clearly displayed.
In case of several menu lines, they are clearly shown	Yes, if they are clearly shown, otherwise No
The menu is designed to suit the target group	Yes, if the menu was presented appropriately for the students of the respective school type and an appropriate font size or pictures made the selection easier, otherwise No
Organic products are used	Yes, if organic products were indicated in the menu, otherwise No
Fair trade products are used	Yes, if fair trade products were indicated in the menu, otherwise No
Sea fish from not overfished stocks	Yes, if the Marine Stewardship Council sign was used in the menu or sea fish from non-overfished stocks was used, such as cod, saithe, herring and mackerel, N/A, if the fish type was not mentioned, No if the fish type was from overfished stocks
Meat from animal friendly husbandry	Yes, if organic meat was indicated in the menu, otherwise No

The meal plans were independently analyzed by two researchers. To ensure data quality, one variable was assessed in two different phrasings. Intrarater reliability demonstrated perfect agreement (Cohen's kappa = 1 p < 0.01) [39]. Interrater reliability between the two raters was substantial, with a Cohen's kappa of 0.67 (p < 0.001), according to the classification by Landis and Koch [39].

### 2.3 Covariates

To account for the possibility, the menus differ by socio-economic status (SES), binomial logistic regression models were estimated.

We implemented the SES in the logistic regression model by adding the Social index for each menu.

To ensure methodologically robust comparability of the index values across both states, the social indices are categorized into three groups: “high,” “middle,” and “low”. The categorization is shown in Table 2. This classification ensures a proportional distribution of the index groups in both states and establishes a comparable basis for further analyses. The index categories were incorporated as dummy variables, representing high and middle socio-economic standards, with low socio-economic standard as reference category.

**Table 2:** Categorization of social index levels by socio-economic status.

Classification	Socio-Economic Standard	Social Index HH	Social Index NRW
1	high	5, 6	1, 2, 3
2	middle	3, 4	4, 5, 6
3	low	1, 2	7, 8, 9

### 2.4 Index development

To provide an overall measure for assessing the effects of the SFS and the co-variables on the menus, as well as to condense the data for more meaningful statistical comparisons, indices were calculated for each sub-category i in Table 1 (mixed diet, ovo-lacto-vegetarian diet, additional criteria for menu planning, and menu criteria). This was done by summing up the criteria i within each sub-category, multiplying the total by 100, and dividing the result by the number of criteria i in the sub-category.

$${Index}_{i}=\frac{{sum\;of\;criteria\;}_{i}}{{number\;of\;criteria}_{i}\;}\;X\;100$$

### 2.5 Statistical analysis

The data collected from the menus were analyzed statistically using IBM SPSS version 30. Descriptive analyses were conducted, with frequencies and percentages calculated in cross-tabulations for nominal categorical variables. Differences between VSFS and OSFS regarding the fulfilment of the DGE-standard requirements were tested using Chi-square or Fisher's exact test, depending on the sample size. Statistical significance was set at p < 0.05. To account for multiple testing, the Bonferroni adjustment was applied, with the alpha level corrected by dividing the p-value by the number of variables in each sub-category.

Binomial logistic regression modelling was used to control for the effects of covariates on the ‘yes’ and ‘no’ categories for each SFS criterion in Table 1. Furthermore, two-step multiple regression models were developed to assess the overall effects on the indexes, examining the influence of SFS and the association of covariates with each index.

## 3 Results

### 3.1 Composition of the sample

**Fig. 1:**
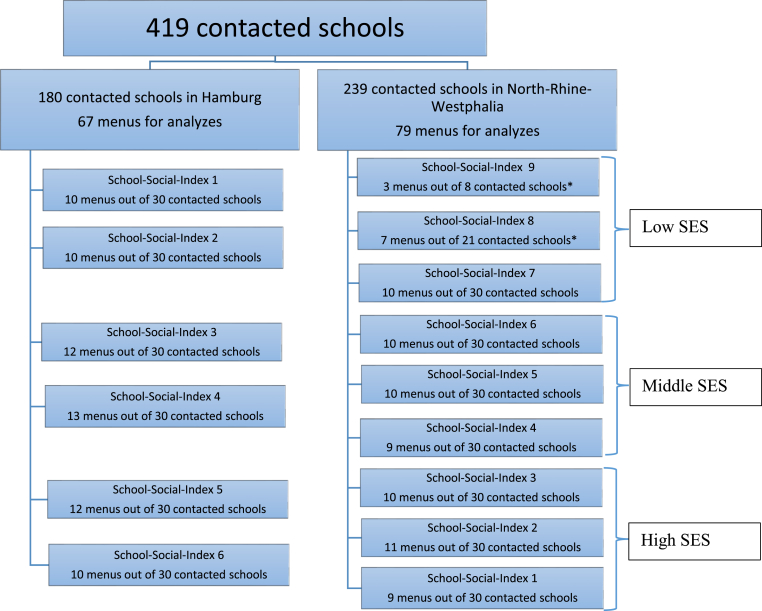
Sample size by federal state and school social Index. ∗All schools for social index 8 and 9 were contacted.

Of the 419 schools contacted, detailed four-week menu plans covering 20 catering days were obtained for 146 school menus. This included 67 menus from Hamburg with OSFS, and 79 menus from North Rhine-Westphalia with VSFS. Among the menus, 114 were from primary schools, and 32 were from secondary schools. The specific distribution of the sample by state and social index is shown in Fig. 1.

### 3.2 Descriptive statistics-fulfillment of the SFS-requirements by OSFS and VSFS

On average, schools achieved approximately 54 % compliance with the SFS standard, corresponding to just over half of the requirements. Schools in Hamburg, where adherence to the SFS is mandatory, demonstrated higher compliance (62.1 %) compared to schools in North Rhine-Westphalia (54.5 %). Table 3 presents descriptive results on the fulfillment of SFS requirements, comparing OSFS and VSFS implementation. After applying the Bonferroni adjustment for multiple comparison, the number of significant differences decreases from 26 to 23. Overall, OSFS menus met SFS criteria more frequently than VSFS menus.

**Table 3:** Fulfillment of the SFS-requirements by menus with OSFS and VSFS in percent.

Requirements of the SFS	OSFS			VSFS				
	Yes (%)	No (%)	N/A (%)	Yes (%)	No (%)	N/A (%)	p-value	Bonferroni's adj.
	
**Design of a health-promoting and sustainable mealfood qualities and frequencies for lunch, mixed diet**								p < 0.004
	
5x grain/-products/potatoes	100.0			88.6	11.4		**0.004∗**	X
of which at least 1x whole grain products (n = 137/OSFS = 67/VSFS = 70)	6.0	94.0		34.3	65.7		**<0.001**	X
of which max. 1x potato products (n = 137/OSFS = 67/VSFS = 70)	97.0	3.0		68.6	31.4		**<0.001**	X
5x vegetables, legumes or salad	95.5	4.5		65.8	34.2		**<0.001**	X
of which at least 2x raw vegetables or salad (n = 116/OSFS = 64/VSFS = 52)	98.4	1.6		84.6	14.4		0.100∗	
of which at least 1x legumes (n = 116/OSFS = 64/VSFS = 52)	56.3	43.8		42.3	57.7		0.135	
At least 2x fruits	100.0			55.7	44.3		**<0.001**	X
of which at least 1x whole fruits (n = 111/OSFS = 67/VSFS = 44)	100.0			100.0				
At least 2x milk and milk products	100.0			100.0				
Max 1x meat and sausages	13.4	86.6		11.4	88.6		0.709	
of which at least 2x lean meat in 20 catering days (n = 18/OSFS = 9/VSFS = 9)	100.0			77.8	22.2		0.471∗	
At least 1x sea fish	62.7	37.3		64.6	35.4		0.815	
of which at least 2x fatty sea fish in 20 catering days (n = 93/OSFS = 42/VSFS = 51)	95.2	4.8		76.5	5.9	17.6	**0.007∗**	
	
**Food qualities and frequencies for a lunch, ovo-lacto-vegetarian diet (n = 109/OSFS = 62/VSFS = 47)**								p < 0.005
	
5x grain/-products/potatoes	100.0			85.1	14.9		**0.002∗**	X
of which at least 1x whole grain products (n = 102/OSFS = 62/VSFS = 40)	9.7	90.3		20.0	80.0		0.139	
of which max. 1x potato products (n = 102/OSFS = 62/VSFS = 40)	21.0	79.0		55.0	45.0		**<0.001**	X
5x vegetables, legumes or salad	98.4	1.6		70.2	29.8		**<0.001**	X
of which at least 2x raw vegetables or salad (n = 94/OSFS = 61/VSFS = 33)	100.0			90.9	9.1		**0.041∗**	
of which at least 1x legumes (n = 94/OSFS = 61/VSFS = 33)	86.9	13.1		48.5	51.5		**<0.001**	X
At least 2 x fruits, nuts and oil seeds	100.0			68.1	31.9		**<0.001**	X
of which at least 1 x whole fruits (n = 94/OSFS = 62/VSFS = 32)	100.0			100.0				
of which at least 1x nuts and oil seeds (n = 94/OSFS = 62/VSFS = 32)		100.0			100.0			
At least 2x milk and milk products	100.0			100.0				
	
**Additional criteria for menu planning**								p < 0.006
	
Ovo-lacto-vegetarian dish available on a daily basis	92.5	7.5		59.5	40.5		**<0.001**	X
Seasonal offer preferred (seasonal fruits and vegetables)	100.0	0.0		83.5	16.5		**<0.001**	X
Regional food products are used	100.0			46.8	53.2		**<0.001**	X
Grain, grain products and potatoes are varied provided	97.9	2.1		88.2	11.8		0.062∗	
Max. 4x fried/breaded products in 20 catering days	95.5	4.4		39.2	60.8		**<0.001**	X
Max. 4x industrial produced meat substitutes in 20 catering days	87.9	12.1		53.2	46.8		**<0.001**	X
Menu cycle is at least four weeks	100.0	0.0		100.0				
Varied dishes	100.0			93.7	6.3		0.062∗	
	
**Menu criteria**								p < 0.004
	
Additives declared	95.5	4.5		75.9	24.1		**<0.001**	X
Allergens declared	100.0			77.2	22.8		**<0.001**	X
Nutrition labeling under compliance with legal requirements			100.0	11.4		88.6	**0.004∗**	X
Nutrient optimized menus emphasized	22.4	77.6		30.4	69.6		0.277	
Unambiguous designation of meals or further explanation	16.4	83.6		51.9	48.1		**<0.001**	X
For meat, sausages and fish the animal species are named	56.7	43.3		36.7	63.3		**0.016**	
If prices are shown, they are clearly displayed			100.0	1.3		98.7	1.000∗	
In case of several menu lines, they are clearly shown (n = 133, OSFS = 64, VSFS = 69))	71.9	28.1		85.5	14.5		0.054	
The menu is designed to suit the target group	16.4	83.6		53.2	46.8		**<0.001**	X
The basis of animal substitute products is clearly labelled	97.0	3.0		55.7	44.3		**<0.001**	X
Organic products are used	100.0			45.6	54.4		**<0,001**	X
Fair trade products are used	7.5	92.5		8.9	91.1		0.759	
Sea fish from not overfished stocks	89.6		10.4	32.9	12.7	54.4	**<0,001∗**	X
Meat from animal friendly husbandry	7.5	92.5		3.8	96.2		0.470∗	

Data analysis using Chi square or Fishers exact test (asterisk∗).

For **mixed diets**, OSFS menus included significantly more *grain products and potatoes* (100 % vs. 89 %, p = 0.004), *vegetables, legumes, and salad* (95 % vs. 66 %, p = 0.002), *fruits* (100 % vs. 56 %, p < 0.001), and *fatty fish* (95 % vs. 77 %, p = 0.007), while offering fewer *potato-based products* (97 % vs. 69 %, p < 0.001). However, VSFS menus contained more *whole grains* (6 % vs. 34 %, p < 0.001).

For **ovo-lacto-vegetarian diets**, OSFS menus included more *grain products and potatoes* (100 % vs. 85 %, p = 0.002), *vegetables, legumes, and salad* (98 % vs. 70 %, p < 0.001), *raw vegetables* (100 % vs. 91 %, p = 0.041), *legumes* (87 % vs. 49 %, p < 0.001), and *fruits and nuts* (100 % vs. 68 %, p < 0.001), but also more *potato products* (21 % vs. 55 %, p < 0.001).

Under **additional menu planning criteria**, all menus followed a minimum four-week cycle and offered variety. However, OSFS menus more often included *vegetarian dishes* (93 % vs. 60 %, p < 0.001), *seasonal* (100 % vs. 84 %, p < 0.001) and *regional products* (100 % vs. 47 %, p < 0.001), *fewer fried items* (96 % vs. 61 %, p < 0.001), and fewer *industrial meat substitutes* (88 % vs. 53 %, p = 0.001).

In the **menu criteria** domain, OSFS menus used more *organic* (100 % vs. 46 %, p = 0.001) and *sustainable fish* products (90 % vs. 33 %, p < 0.001), and provided better ingredient transparency: *animal species* (57 % vs. 37 %, p < 0.001), *basis of animal substitutes* (97 % vs. 56 %, p < 0.001), *additives* (96 % vs. 76 %, p < 0.001), and *allergens* (100 % vs. 77 %, p = 0.001). However, VSFS menus were more often *tailored to the target audience* (16 % vs. 53 %, p < 0.001), i*ncluded nutritional information* (0 % vs. 11.4 %%, p = 0.004) and had *clearer menu descriptions* (16 % vs 52 %, p = 0.001).

### 3.3 Association between school social index and sub-category indexes

Two-step multiple regression models were performed to assess the effect of the SFS on the sub-category indexes outlined in Table 1. Stepwise multiple regression was used to determine the impact of the covariate, the school social index, on the sub-category indexes. The results of the two-step regression models are presented in Supplementary Table 2.

Overall, a low proportion of the dependent variables’ variance is explained by the **mixed diet** and the **menu criteria** models. For the **ovo-lacto-vegetarian diet** model a medium variance of 17 % for the Step 1 model and 19.9 % for the Step 2 model is explained according to Cohen (1988) [40]. The **additional menu planning** models explain with 57.6 % (Step 1) and 58.5 % (Step 2) a strong variance.

As shown by the Step 1 models, all four indices are significantly influenced by the OSFS/VSFS variable, and the fulfillment of the indices is higher when the SFS is applied mandatorily.

However, adding the school social index in Step 2 does not significantly improve model fit. Although some trends suggest better outcomes with higher social indices for most models (except the vegetarian diet), none of these effects are statistically significant.

### 3.4 Association between school social index and the SFS criteria

Binomial logistic regressions were performed to determine the association between the covariate, school social index, and all observable SFS criteria listed in Table 1. The association of the covariate with the individual menu criteria is presented in Supplementary Table 3.

Overall, the influence of the social index on the menu criteria was not consistent across all models. According to Muijs (2011) [41], model fit improvements, as indicated by Nagelkerke's R^2^, ranged from poor (<0.1) in 9 models, to modest (0.1–0.3) in 18 models, to moderate (0.3–0.5) in 8 models, and strong (R^2^ > 0.5) in 3 models.

For **mixed diets**, six models showed statically significant associations: *grain products* (χ^2^(3) = 13.227, p = 0.004), *whole grain* (χ^2^(3) = 18.958, p < 0.001), *potato products* (χ^2^(3) = 22.056, p < 0.001), *vegetables, legumes or salad* (χ^2^(3) = 23.262, p = 0.006), *raw vegetables or salad* (χ^2^(3) = 12.406, p < 0.006) and *fruits* (χ^2^(3) = 53.703, p = 0.001). The five **ovo-lacto vegetarian** models for the criteria *grain products* (χ^2^(3) = 13.263, p = 0.004), *potato products* (χ ^2^(3) = 15.953, p = 0.001), *vegetables, legumes or salad* (χ ^2^(3) = 23.275, p < 0.001), *legumes* (χ^2^(3) = 18.415, p < 0.001) and *fruits, nuts and oil seeds* (χ ^2^(3) = 29.203, p < 0.001) are significant. Among the **additional menu planning** criteria, six models were significant: *ovo-lacto-vegetarian dish* (χ^2^(3) = 24.389, p < 0.001), *seasonal offer* (χ^2^(3) = 18.369, p < 0.001), *regional food products* (χ ^2^(3) = 78.984, p < 0.001), *use of fried and breaded products* (χ^2^(3) = 59.142, p < 0.001), *industrial meat substitutes* (χ^2^(3) = 25.279, p < 0.001) and *varied dish* (χ^2^(3) = 12.359, p = 0.006). The nine significant binomial logistic regression models for the **menu criteria** are: *additives declared* (χ^2^(3) = 17.248, p < 0.001), *allergens declared* (χ^2^(3) = 28.424, p < 0.001), *nutrition labeling* (χ^2^(3) = 11.885, p = 0.008), *animal species* (χ^2^(3) = 11.199, p = 0.011), *several clear menu lines* (χ^2^(3) = 12.078, p = 0.007), *target group designed menu* (χ ^2^(3) = 23.211, p < 0.001), *organic products* (χ ^2^(3) = 74.722, p < 0.001), and ‘sustainable fish’ (χ ^2^(3) = 62.842, p < 0.001).

In the majority, the obligatory OSFS had a positive effect on the fulfilment of the menu criteria. In the significant models, the influence of the SFS was significant in fifteen models. In ten cases, the OSFS was associated with better fulfillment of the respective criteria. If the SFS was obligatory, the odds of fulfilling the **mixed diet** criteria increased for *potato products* (OR = 14.85, p < 0.001), *vegetables, legumes or salad* (OR = 11.05, p < 0.001) and for *raw vegetables* (OR = 11.91, p = 0.023). Whereas grain products increase in VSFS menus (OR = 0.123, p < 0.001). For the **ovo-lacto-vegetarian diet**, *vegetables, legumes and salad* increased (OR = 27.41, p = 0.002) and the use of *legumes* (OR = 7.05, p = 0.001) when the OSFS is used. Whereas *potato products* are less present in VSFS (OR = 0.202, p < 0.001).

Regarding **additional menu planning** criteria OFSF menus showed greater inclusion of *ovo-lacto vegetarian dishes* (OR = 8.41, p < 0.001), less frequent use of *fried and breaded products* (OR = 33.99, p = 0.001) and *industrial meat substitutes* (OR = 6.92, p < 0.001), relative to VSFS menus. Furthermore, OSFS menus were more likely to meet specific labeling and sustainability criteria: *additives are declared* (OR = 7.71, p = 0.002), the *animal species is named* (OR = 2.41, p = 0.012) and more *sustainable fish* is used (OR = 21.97, p < 0.001). In contrast, VSFS menus had higher odds for a *clear meal description* (OR = 0.183, p < 0.001) and offering *menus designed for the target group* (OR = 0.169, p < 0.001).

There is no clear association between **school social indices** and SFS criteria. Only two criteria showed significant differences. The presence of *clear menu lines* increased with higher socioeconomic status (OR = 3.539, *p* = 0.020) to low SES, while the use of *fatty fish* was more common in menus from schools in lower socioeconomic areas (OR = 0.258, *p* = 0.018), compared to those in high SES areas.

## 4 Discussion

This study aimed to assess whether students from all socioeconomic backgrounds receive healthy school lunches by analyzing adherence to SFS requirements and comparing OSFS versus VSFS implementation, as well as the influence of school social indices.

Descriptive analysis revealed that most SFS requirements were only partially met, regardless of implementation type on average only 54 % of the SFS are met by school menus. However, OSFS menus generally aligned better with the standards (62 % versus 48 %).

OSFS menus for mixed and ovo-lacto-vegetarian diet featured healthier patterns, with more grain products and potatoes, vegetables, legumes, fruits and fewer fried or industrial products. Mixed diets additionally included more fatty fish and fewer potato-based items like fries or mashed potatoes. In contrast, VSFS menus included more whole-grain products. Ovo-lacto-vegetarian OSFS menus include additionally more raw vegetables. Conversely, potato products appeared less frequently in vegetarian VSFS menus.

These findings suggest mandatory implementation is more effective than voluntary adoption in meeting the SFS requirements. This aligns with previous findings from our nationwide study [29], supporting the broader observation that voluntary adherence to policies in the food and beverage industry often has minimal impact on public health promotion. Such an approach may be unsuitable for driving meaningful changes or improvements in consumer behaviour [42].

In general, mandatory policies that limit individual freedom of choice must be evaluated and balanced against their proven ability to drive meaningful and desirable changes. Such changes must be supported by scientific evidence. To ensure all students have access to healthy and sustainable meals, organizations like the German School Catering Network (DNSV) and the German Obesity Society advocate for compulsory SFS implementation [43,44]. Furthermore, more than 60 % of German consumers support food policy interventions and would welcome government measures to provide healthier food options [45].

Despite this, SFS implementation remains voluntary in most German federal states due to policy decentralization, with only five states mandating it. Justifying these mandates requires scientific proof of their added value—something this study provides by comparing the effects of mandatory and voluntary policies on school meal quality.

Half of European countries have already adopted binding SFSs [16]. Still, various studies conducted in Europe and the Western Pacific highlight a generally low willingness to comply with such policies [46]. These findings underline the need not only for mandatory SFS adoption but also for regular monitoring to ensure consistent implementation, as both groups in this study showed gaps in meeting the standards.

To obtain SFS certification, schools must achieve at least 60 % compliance across various quality domains [47]. While our study does not encompass all domains, the findings already indicate that the minimum threshold is only narrowly met, with 62 % compliance in the analyzed criteria. Consequently, to enhance both compliance with and participation in the SFS, additional aspects such as stakeholder engagement warrant further investigation. Existing research suggests that comprehensive school food policies which actively involve key stakeholders—including students, school principals, parents, teachers, food service providers, and citizen committees—as well as supplementary educational campaigns, can increase acceptance, strengthen implementation, and ultimately improve the quality of school meals [[48], [49], [50]]. Further studies are required to examine these dynamics in the context of both voluntary and mandatory SFS implementation. Significant differences in the fulfillment of SFS criteria across school social index categories were observed only in two cases: menus from higher socio-economic status (SES) schools featured clearer menu designs, while fatty fish was served more frequently in lower SES schools. These findings align with a Canadian study showing that schools in socioeconomically disadvantaged areas face greater exposure to unhealthy food options around schools [31], positioning SFS-compliant school lunches as a valuable healthy alternative in such settings.

The European Child Guarantee urges member states to provide one healthy school meal per day and ensure effective access to nutritious food for children in need [51]. Since school SES had little overall impact on menu quality, SFS-aligned meals appear to be a viable tool for offering all students—regardless of background—equal access to healthy nutrition. These results support the introduction of free school meals, particularly benefiting children from low-income families by helping prevent diet-related diseases and promoting overall child health [52,53]. The results support the hypothesis that the SFS has its impact regardless of socioeconomic factors when implemented mandatorily.

This study has several limitations. First, several SFS criteria could not be assessed, as they were not observable from the school menus. These include preparation guidelines (e.g., reduced fat, sugar, and salt) and food service aspects like portion sizes and access to drinking water. Studies have shown that school food interventions can lead to modifications in school meal recipes. For instance, evidence from elementary schools in the United States indicates that, following such interventions, school meals contained lower levels of salt, total fat, and saturated fat, while still meeting recommendations for calories and essential nutrients [54]. In line with these findings, Micha et al. [55], in a review of studies conducted in high-income countries, reported a reduction in total fat, saturated fat, and salt content associated with the implementation of SFSs, whereas overall caloric intake remained unchanged. Future research using alternative methods, like recipe analysis and site visits in kitchen and schools is needed to evaluate these elements. Second, as the first exploratory analysis of its kind in this region, the study used a convenience sample, which limited the ability to conduct power, effect size, or sample size calculations. While significant results were observed, some effects may have gone undetected due to limited statistical power. Non-responder bias is another potential limitation, given the reliance on written participation requests.

Moreover, further research is needed to identify strategies that can enhance compliance and participation in SFS, ensuring that potential health benefits reach all students.

Nevertheless, the study design was appropriate for its aim: to explore the fulfillment of SFS criteria by comparing mandatory versus voluntary implementation across social index categories. The findings provide a valuable starting point for informing the discussion about whether mandatory SFS implementation is necessary to improve school food quality for students from all socio-economic backgrounds. The results also highlight the need to strengthen adherence to SFS standards, regardless of implementation type.

## Ethical statement

This work did not involve human subjects or animals in its research. Neither ethical approval nor individual consent was applicable.

## Declaration of generative AI and AI-assisted technologies

During the preparation of this work the authors used ChatGPT (GPT-4 by OpenAI) in terms of language and phrasing as a writing assistant. The authors reviewed and edited the content as needed and take full responsibility for the content of the publication.

## Funding

This research did not receive any specific grant from funding agencies in the public, commercial, or not-for-profit sectors.

## Declaration of competing interest

The authors declare that they have no known competing financial interests or personal relationships that could have appeared to influence the work reported in this paper.
