# Supplementary material for: Equal school meals for all - differentiation of school meals according to school social index
Source: Public Health Pract (Oxf). 2025 Dec 11;11:100693. doi: 10.1016/j.puhip.2025.100693 (PMC12765146; doi:10.1016/j.puhip.2025.100693)
Supplement: Multimedia component 1 [file mmc1.docx]

Supplementary table 1: Requirements of the 2023 SFS and the integration in the analyses

| **Requirements** | **Integration in analyses** |
| --- | --- |
| **Development of quality school meals** | |
| A catering concept is in place |  |
| All stakeholders are involved |  |
| A catering commissioner exists |  |
| Catering staff receive continuous training |  |
| Ergonomic workplaces and workflows are in place |  |
| Employees are valued |  |
| Feedback on the food offerings is regularly collected, evaluated, and used to develop actions. |  |
| **Design of a health-promoting and sustainable meal**  *food qualities and frequencies for lunch, mixed diet, 5 catering days* | |
| 5x (1x daily) grain/ -products/ potatoes | X |
| of which at least 1x whole grain products | X |
| of which max. 1x potato products | X |
| 5x (1x daily) vegetables, legumes or salad | X |
| of which at least 2x raw vegetables or salad | X |
| of which at least 1x legumes | X |
| At least 2 x fruits | X |
| of which at least 1 x whole fruits | X |
| At least 2x milk and milk products | X |
| Max 1x meat and sausages | X |
| of which at least 2x lean meat in 20 catering days | X |
| At least 1x sea fish | X |
| of which at least 2x fatty sea fish in 20 catering days | X |
| Rapeseed oil is a standard oil |  |
| Beverage are available at any time (water, herbs- and fruit tea) |  |
| *Food qualities and frequencies for a lunch, ovo-lacto-vegetarian diet in 5 catering days* | |
| 5x (1x daily) grain/ -products/ potatoes | X |
| of which at least 1x whole grain products | X |
| of which max. 1x potato products | X |
| 5x (1x daily) vegetables, legumes or salad | X |
| of which at least 2x raw vegetables or salad | X |
| of which at least 1x legumes | X |
| At least 2 x fruits, nuts and oil seeds | X |
| of which at least 1 x whole fruits | X |
| of which at least 1x nuts and oil seeds | X |
| At least 2x milk and milk products | X |
| Rapeseed oil is a standard oil |  |
| Beverage are available at any time (water, herbs- and fruit tea) |  |
| **Additional criteria for menu planning** | |
| Ovo-lacto-vegetarian dish available on a daily basis | X |
| Seasonal offer preferred (seasonal fruits and vegetables) | X |
| Regional food products are used | X |
| Grain, grain products and potatoes are varied provided | X |
| Max. 4x fried/ breaded products in 20 days | X |
| Max. 4x industrial produced meat substitutes in 20 days | X |
| Menu cycle is at least four weeks | X |
| The dishes are varied | X |
| Drinks are always available |  |
| Participation in meals is possible in case of food intolerances like allergies |  |
| The students wished and suggestions are considered in the menu planning as far as possible |  |
| Culture-specific, regional and religious eating habits are taken into account in planning |  |
| **Criteria of the use of convenience food in mass catering** | |
| Products without palm (kernel) fat, palm (kernel) oil or coconut fat are preferred |  |
| Unprocessed or low processed products, like fresh or frozen vegetables and fruits, meat or fish, are preferred for further processing on site |  |
| High processed products are always combined or complemented with low processed products/components |  |
| Products with a low content of sugar, fat, saturated fat and / or salt and a low energy density are selected |  |
| **Menu criteria** | |
| The current menu is accessible in advance on a regular and barrier-free basis |  |
| Additives declared | X |
| Allergens declared | X |
| Nutrition labeling under compliance with legal requirements | X |
| Nutrient optimized menus emphasized | X |
| Unambiguous designation of meals or further explanation | X |
| For meat, sausages and fish the animal species are named | X |
| The basis of animal substitute products (meat, fish, egg, milk, milk-products) is clearly labelled | X |
| If prices are shown, they are clearly displayed | X |
| In case of several menu lines, they are clearly shown | X |
| The menu is designed to suit the target group | X |
| Organic products are used | X |
| Fair trade products are used | X |
| Sea fish from not overfished stocks | X |
| Meat from animal friendly husbandry | X |
| Environmentally friendly packaging is preferred for all foods |  |
| Frist –in-first out principle applies |  |
| Recipes, with preparation instructions if required, are used |  |
| Fat is used consciously |  |
| Sugar is used sparingly |  |
| Iodised salt is used, it is salted sparingly |  |
| Herbs (fresh, frozen, dried) and spices are used in a variety of ways. |  |
| Nutrient-preserving and low-fat cooking methods are used. |  |
| Cooking periods are kept as long as necessary and as short as possible |  |
| Keeping heated food warm for a maximum of three hours |  |
| The warm-keeping temperature of heated food is at least 60°C |  |
| Chilled food is stored at a maximum of 7°C |  |
| Resource-efficient kitchen appliances are used |  |
| Appliances are only turned on during operating times |  |
| Proper timing between kitchen and serving is realized |  |
| Serving staff is informed in detail about the current menu |  |
| Students are given opportunities to influence portion sizes |  |
| Students are given friendly advice when ordering and choosing food. The principle of nudging is considered |  |
| Questions about a wholesome diet and food intolerances are answered |  |
| Returned dishes are recorded separately by meal and component and the outcomes are used for future menu planning |  |
| Unavoidable waste is recycled for energy utilization |  |
| Attention is paid to the use of environmentally friendly cleaning agents |  |
| Dosing aids are used |  |
| Hygiene requirements are observed |  |
| **Living environment** | |
| Sufficient and, if necessary, staggered break times are planned |  |
| Enable eating and drinking in an age-appropriate and appealing ambience |  |

Supplementary table 2 Results of two-step multiple regression analyses

|  | B | SE B | β | p-value |
| --- | --- | --- | --- | --- |
| **Index mixed diet** |  |  |  |  |
| **Step 1** |  |  |  |  |
| Constant | 66.99 | 1.87 |  |  |
| SFS (OSFS vs VSFS) | 10.16 | 2.76 | 0.29 | **<0.001** |
| **Step 2** |  |  |  |  |
| Constant | 65.87 | 2.99 |  |  |
| SFS (OSFS vs VSFS) | 10.19 | 2.78 | 0.29 | **<0.001** |
| Social index (high vs low) | 0.18 | 3.53 | 0.005 | 0.961 |
| Social index (middle vs low) | 2.79 | 3.48 | 0.78 | 0.424 |
| R²=0.086 for step 1; R²_Change_= 0.006 for step 2, p_Change_= 0.641 |  |  |  |  |
| **Index ovo-lacto-vegetarian diet (19,9%)** |  |  |  |  |
| **Step 1** |  |  |  |  |
| Constant | 59.94 | 2.04 |  |  |
| SFS (OSFS vs VSFS) | 12.68 | 2.70 | 0.41 | **<0.001** |
| **Step 2** |  |  |  |  |
| Constant | 62.6 | 2.92 |  |  |
| SFS (OSFS vs VSFS) | 12.47 | 2.68 | 0.41 | **<0.001** |
| Social index (high vs low) | -6.51 | 3.39 | -0.2 | 0.058 |
| Social index (middle vs low) | -3.77 | 3.25 | -0.12 | 0.249 |
| R²=0.171 for step 1; R²_Change_= 0.028 for step 2, p_Change_= 0.136 |  |  |  |  |
| **Index additional criteria for menu planning (58,5%)** |  |  |  |  |
| **Step 1** |  |  |  |  |
| Constant | 71.84 | 1.22 |  |  |
| SFS (OSFS vs VSFS) | 25.13 | 1.80 | 0.76 | **<0.001** |
| **Step 2** |  |  |  |  |
| Constant | 70.44 | 1.94 |  |  |
| SFS (OSFS vs VSFS) | 25.15 | 1.78 | 0.76 | **<0.001** |
| Social index (high vs low) | 0.33 | 2.3 | 0.01 | 0.8886 |
| Social index (middle vs low) | 3.36 | 2.26 | 0.1 | 0.139 |
| R²=0.576 for step 1; R²_Change_= 0.009 for step 2, p_Change_= 0.230 |  |  |  |  |
| **Index menu criteria** |  |  |  |  |
| **Step 1** |  |  |  |  |
| Constant | 43.06 | 1.88 |  |  |
| SFS (OSFS vs VSFS) | 5.98 | 2.69 | 0.19 | **0.028** |
| **Step 2** |  |  |  |  |
| Constant | 39.60 | 2.90 |  |  |
| SFS (OSFS vs VSFS) | 6.19 | 2.68 | 0.19 | **0.022** |
| Social index (high vs low) | 2.63 | 3.43 | 0.08 | 0.445 |
| Social index (middle vs low) | 6.40 | 3.33 | 0.20 | 0.056 |
| R²=0.062 for step 1; R²_Change_= 0.027 for step 2, p_Change_= 0.149 |  |  |  |  |

Supplementary table 3: Results of binomial logistic regression analysis

|  | Odds ratio | 95% confidence intervall | p-value |
| --- | --- | --- | --- |
| **Mixed diet** | | | |
| **5x grain/ -products/ potatoes** |  |  |  |
| Constant | 9.00 |  |  |
| SFS (OSFS vs VSFS) | 202160001.12 | 0.000 | 0.997 |
| Social index (high vs low) | 0.533 | 0.093-3.069 | 0.481 |
| Social index (middle vs low) | 1.556 | 0.201-12.053 | 0.672 |
| χ²(3)=13.227, **p<0.004**, Nagelkerke’s *R*²=0.234 |  |  |  |
| **of which at least 1x whole grain products** (n=137) |  |  |  |
| Constant | 0.393 |  |  |
| SFS (OSFS vs VSFS) | 0.123 | 0.040-0.378 | **<0.001** |
| Social index (high vs low) | 1.418 | 0.432-4.652 | 0.564 |
| Social index (middle vs low) | 1.486 | 0.470-4.699 | 0.500 |
| χ²(3)=18.958, **p<0.001**, Nagelkerke’s *R*²=0.203 |  |  |  |
| **of which max. 1x potato products** (n=137) |  |  |  |
| Constant | 2.312 |  |  |
| SFS (OSFS vs VSFS) | 14.854 | 3.330-66.262 | **<0.001** |
| Social index (high vs low) | 0.977 | 0.281-3.402 | 0.971 |
| Social index (middle vs low) | 0.883 | 0.266-2.930 | 0.883 |
| χ²(3)=22.056, p**<0.001**, Nagelkerke’s *R*²=0.246 |  |  |  |
| **5x vegetables, legumes or salad** |  |  |  |
| Constant | 2.218 |  |  |
| SFS (OSFS vs VSFS) | 11.047 | 3.164-38.566 | **<0.001** |
| Social index (high vs low) | 0.664 | 0.219-2.015 | 0.470 |
| Social index (middle vs low) | 1.043 | 0.334-3.253 | 0.943 |
| χ²(3)=23.262, **p<0.001**, Nagelkerke’s *R*²=0.231 |  |  |  |
| **of which at least 2x raw vegetables or salad** (n=116) |  |  |  |
| Constant | 4.309 |  |  |
| SFS (OSFS vs VSFS) | 11.914 | 1.416-100.270 | **0.023** |
| Social index (high vs low) | 0.704 | 0.144-3.454 | 0.666 |
| Social index (middle vs low) | 4.917 | 0.466-51.883 | 0.185 |
| χ²(3)=12.406, **p=0.006**, Nagelkerke’s *R*²=0.241 |  |  |  |
| **of which at least 1x legumes** (n=116) |  |  |  |
| Constant | 0.399 |  |  |
| SFS (OSFS vs VSFS) | 1.827 | 0.860-3.883 | 0.117 |
| Social index (high vs low) | 2.266 | 0.860-5.968 | 0.098 |
| Social index (middle vs low) | 2.253 | 0.886-5.725 | 0.088 |
| χ²(3)=5.954, p=0.114, Nagelkerke’s *R*²=0.067 |  |  |  |
| **At least 2 x fruits** |  |  |  |
| Constant | 1.857 |  |  |
| SFS (OSFS vs VSFS) | 1276129293.423 | 0.000 | 0.997 |
| Social index (high vs low) | 0.503 | 0.156-1.623 | 0.250 |
| Social index (middle vs low) | 0.704 | 0.219-2.266 | 0.556 |
| χ²(3)=53.703, **p<0.001**, Nagelkerke’s *R*²=0.461 |  |  |  |
| **of which at least 1 x whole fruits** (n=111) | No differences, the criteria is 100% fulfilled for all menus | | |
| **At least 8x milk and milk products** | No differences, the criteria is 100% fulfilled for all menus | | |
| **Max 1x meat and sausages** |  |  |  |
| Constant | 0.163 |  |  |
| SFS (OSFS vs VSFS) | 1.173 | 0.432-3.186 | 0.754 |
| Social index (high vs low) | 0.358 | 0.083-1.534 | 0.166 |
| Social index (middle vs low) | 1.117 | 0.362-3.442 | 0.847 |
| χ²(3)=3.481, p=0.323, Nagelkerke’s *R*²=0.045 |  |  |  |
| **of which at least 2x lean meat in 20 catering days** (n=18) |  |  |  |
| Constant | 2.000 |  |  |
| SFS (OSFS vs VSFS) | 350974860.063 | 0.000 | 0.999 |
| Social index (high vs low) | 0.500 | 0.013-19.562 | 0.711 |
| Social index (middle vs low) | 281752486.091 | 0.000 | 0.999 |
| χ²(3)=5.966, p=0.113, Nagelkerke’s *R*²=0.562 |  |  |  |
| **At least 1x sea fish** |  |  |  |
| Constant | 1.137 |  |  |
| SFS (OSFS vs VSFS) | 0.945 | 0.476-1.876 | 0.871 |
| Social index (high vs low) | 1.652 | 0.709-3.854 | 0.245 |
| Social index (middle vs low) | 2.200 | 0.939-5.154 | 0.069 |
| χ²(3)=3.424, p=0.331, Nagelkerke’s *R*²=0.032 |  |  |  |
| **of which at least 2x fatty sea fish in 20 catering days** (n=84) |  |  |  |
| Constant | 1329254227.852 |  |  |
| SFS (OSFS vs VSFS) | 1.642 | 0.249-10.839 | 0.606 |
| Social index (high vs low) | 0.000 | 0.000 | 0.999 |
| Social index (middle vs low) | 0.000 | 0.000 | 0.999 |
| χ²(3)=2.473, p=0.480, Nagelkerke’s *R*²=0.080 |  |  |  |
| **Ovo-lacto-vegetarian diet (n=109)** | | | |
| **5x grain/ -products/ potatoes** |  |  |  |
| Constant | 12.00 |  |  |
| SFS (OSFS vs VSFS) | 275468770.02 | 0.00-0.00 | 0.998 |
| Social index (high vs low) | 0.361 | 0.033-3.96 | 0.405 |
| Social index (middle vs low) | 0.417 | 0.04-4.53 | 0.472 |
| χ²(3)=13.263, **p=0.004**, Nagelkerke’s *R*²=0.302 |  |  |  |
| **of which at least 1x whole grain products** (n=102) |  |  |  |
| Constant | 0.39 |  |  |
| SFS (OSFS vs VSFS) | 0.42 | 0.13-1.34 | 0.143 |
| Social index (high vs low) | 0.58 | 0.14-2.33 | 0.440 |
| Social index (middle vs low) | 0.47 | 0.12-1.87 | 0.283 |
| χ²(3)=3.391, p<0.335, Nagelkerke’s *R*²=0.059 |  |  |  |
| **of which max. 1x potato products** (n=102) |  |  |  |
| Constant | 2.171 |  |  |
| SFS (OSFS vs VSFS) | 0.202 | 0.082-0.496 | **<0.001** |
| Social index (high vs low) | 0.341 | 0.107-1.084 | 0.068 |
| Social index (middle vs low) | 0.563 | 0.197-1.608 | 0.284 |
| χ²(3)=15.953, **p=0.001**, Nagelkerke’s *R*²=0.200 |  |  |  |
| **5x vegetables, legumes or salad** |  |  |  |
| Constant | 3.625 |  |  |
| SFS (OSFS vs VSFS) | 27.412 | 3.383-222.138 | **0.002** |
| Social index (high vs low) | 0.319 | 0.066-1.532 | 0.154 |
| Social index (middle vs low) | 1.041 | 0.195-5.577 | 0.962 |
| χ²(3)=23.275, **p<0.001**, Nagelkerke’s *R*²=0.349 |  |  |  |
| **of which at least 2x raw vegetables or salad** (n=94) |  |  |  |
| Constant | 10.000 |  |  |
| SFS (OSFS vs VSFS) | 167141094.543 | 0.000 | 0.997 |
| Social index (high vs low) | 0.700 | 0.037-13.179 | 0.812 |
| Social index (middle vs low) | 1.300 | 0.072-23.434 | 0.859 |
| χ²(3)=6.636, p=0.084, Nagelkerke’s *R*²=0.277 |  |  |  |
| **of which at least 1x legumes** (n=94) |  |  |  |
| Constant | 0.637 |  |  |
| SFS (OSFS vs VSFS) | 7.050 | 2.522-19.704 | **<0.001** |
| Social index (high vs low) | 3.063 | 0.739-12.688 | 0.123 |
| Social index (middle vs low) | 1.335 | 0.422-4.223 | 0.623 |
| χ²(3)=18.415, **p<0.001**, Nagelkerke’s *R*²=0.259 |  |  |  |
| **At least 2 x fruits, nuts and oil seeds** |  |  |  |
| Constant | 3.333 |  |  |
| SFS (OSFS vs VSFS) | 751227214.373 | 0.000 | 0.997 |
| Social index (high vs low) | 0.500 | 0.097-2.577 | 0.407 |
| Social index (middle vs low) | 0.600 | 0.119-3.023 | 0.537 |
| χ²(3)=29.203, **p<0.001**, Nagelkerke’s *R*²=0.426 |  |  |  |
| **of which at least 1 x whole fruits** (n=94) | No differences, the criteria is 100% fulfilled for all menus | | |
| **of which at least 1 x nuts and oil seeds** (n=94) | No differences, the criteria is 100% not fulfilled for all menus | | |
| **At least 2x milk and milk products** | No differences, the criteria is 100% fulfilled for all menus | | |
| **Additional criteria for menu planning** | | | |
| **Ovo-lacto-vegetarian dish available on a daily basis** |  |  |  |
| Constant | 1.925 |  |  |
| SFS (OSFS vs VSFS) | 8.408 | 3.034-23.304 | **<0.001** |
| Social index (high vs low) | 0.569 | 0.199-1.627 | 0.292 |
| Social index (middle vs low) | 0.857 | 0.296-2.484 | 0.776 |
| χ²(3)=24.389, **p<0.001**, Nagelkerke’s *R*²=0.227 |  |  |  |
| **Seasonal offer preferred (seasonal fruits and vegetables)** |  |  |  |
| Constant | 3.000 |  |  |
| SFS (OSFS vs VSFS) | 332837334.998 | 0.000 | 0.997 |
| Social index (high vs low) | 2.083 | 0.483-8.992 | 0.325 |
| Social index (middle vs low) | 2.167 | 0.503-9.332 | 0.299 |
| χ²(3)=18.369, **p<0.001**, Nagelkerke’s *R*²=0.262 |  |  |  |
| **Regional food products are used** |  |  |  |
| Constant | 0.818 |  |  |
| SFS (OSFS vs VSFS) | 2312840902.630 | 0.000 | 0.996 |
| Social index (high vs low) | 0.389 | 0.114-1.323 | 0.131 |
| Social index (middle vs low) | 2.852 | 0.879-9.255 | 0.081 |
| χ²(3)=78.984, **p<0.001**, Nagelkerke’s *R*²=0.598 |  |  |  |
| **Grain, grain products and potatoes are varied provided** |  |  |  |
| Constant | 675726415.940 |  |  |
| SFS (OSFS vs VSFS) | 16322891.714 | 0.000 | 0.997 |
| Social index (high vs low) | 1.170 | 0.000 | 1.000 |
| Social index (middle vs low) | 0.000 | 0.000 | 0.998 |
| χ²(3)=3.192, p=0.363, Nagelkerke’s *R*²=0.275 |  |  |  |
| **Max. 4x fried/ breaded products in 20 catering days** |  |  |  |
| Constant | 0.542 |  |  |
| SFS (OSFS vs VSFS) | 33.993 | 9.748-118.547 | **<0.001** |
| Social index (high vs low) | 1.456 | 0.489-4.333 | 0.500 |
| Social index (middle vs low) | 1.096 | 0.372-3.230 | 0.868 |
| χ²(3)=59.142, **p<0.001**, Nagelkerke’s *R*²=0.459 |  |  |  |
| **Max. 4x industrial produced meat substitutes in 20 catering days** |  |  |  |
| Constant | 0.702 |  |  |
| SFS (OSFS vs VSFS) | 6.915 | 2.870-16.665 | **<0.001** |
| Social index (high vs low) | 2.563 | 0.942-6.976 | 0.065 |
| Social index (middle vs low) | 1.447 | 0.563-3.721 | 0.443 |
| χ²(3)=25.279, **p<0.001**, Nagelkerke’s *R*²=0.225 |  |  |  |
| **Menu cycle is at least four weeks** | No differences, the criteria is 100% fulfilled for all menus | | |
| **Varied dishes** |  |  |  |
| Constant | 19.000 |  |  |
| SFS (OSFS vs VSFS) | 94115213.917 | 0.000 | 0.997 |
| Social index (high vs low) | 0.329 | 0.034-3.187 | 0.337 |
| Social index (middle vs low) | 39077114.114 | 0.000 | 0.997 |
| χ²(3)=12.359, **p=0.006**, Nagelkerke’s *R*²=0.315 |  |  |  |
| **Menu criteria** | | | |
| **Additives declared** |  |  |  |
| Constant | 1.407 |  |  |
| SFS (OSFS vs VSFS) | 7.705 | 2.107-28.176 | **0.002** |
| Social index (high vs low) | 3.129 | 0.961-10.187 | 0.058 |
| Social index (middle vs low) | 3.298 | 1.018-10.690 | 0.047 |
| χ²(3)=17.248, **p<0.001**, Nagelkerke’s *R*²=0.195 |  |  |  |
| **Allergens declared** |  |  |  |
| Constant | 1.500 |  |  |
| SFS (OSFS vs VSFS) | 530079301.882 | 0.000 | 0.997 |
| Social index (high vs low) | 3.200 | 0.859-11.917 | 0.083 |
| Social index (middle vs low) | 3.333 | 0.897-12.383 | 0.072 |
| χ²(3)=28.424, **p<0.001**, Nagelkerke’s *R*²=0.336 |  |  |  |
| **Nutrition labeling under compliance with legal requirements** |  |  |  |
| Constant | 5.667 | 0.000 | 0.997 |
| SFS (OSFS vs VSFS) | 212120647.267 | 0.276-8.482 | 0.627 |
| Social index (high vs low) | 1.529 | 0.287-8.794 | 0.596 |
| Social index (middle vs low) | 1.588 |  |  |
| χ²(3)=11.885, **p=0.008**, Nagelkerke’s *R*²=0.211 |  |  |  |
| **Nutrient optimized menus emphasized** (n=146) |  |  |  |
| Constant | 0.527 |  |  |
| SFS (OSFS vs VSFS) | 0.650 | 0.307-1.378 | 0.261 |
| Social index (high vs low) | 0.695 | 0.271-1.783 | 0.449 |
| Social index (middle vs low) | 0.857 | 0.347-2.118 | 0.738 |
| χ²(3)=1.778, p=0.620, Nagelkerke’s *R*²=0.018 |  |  |  |
| **Unambiguous designation of meals or further explanation** |  |  |  |
| Constant | 0.751 |  |  |
| SFS (OSFS vs VSFS) | 0.183 | 0.083-0.402 | **0.001** |
| Social index (high vs low) | 1.631 | 0.627-4.241 | 0.316 |
| Social index (middle vs low) | 1.616 | 0.629-4.155 | 0.319 |
| χ²(3)=22.182, **p<0.001**, Nagelkerke’s *R*²=0.194 |  |  |  |
| **For meat, sausages and fish the animal species are named** |  |  |  |
| Constant | 0.300 |  |  |
| SFS (OSFS vs VSFS) | 2.411 | 1.217-4.776 | **0.012** |
| Social index (high vs low) | 2.022 | 0.835-4.897 | 0.119 |
| Social index (middle vs low) | 2.712 | 1.133-6.491 | 0.025 |
| χ²(3)=11.199, **p=0.011**, Nagelkerke’s *R*²=0.099 |  |  |  |
| **If prices are shown, they are clearly displayed** |  |  |  |
| Constant | 0.000 |  |  |
| SFS (OSFS vs VSFS) | 0.000 | 0.000 | 0.997 |
| Social index (high vs low) | 24195635.685 | 0.000 | 0.998 |
| Social index (middle vs low) | 0.900 | 0.000 | 1.000 |
| χ²(3)=3.261, p=0.353, Nagelkerke’s *R*²=0.281 |  |  |  |
| **In case of several menu lines, they are clearly shown** (n=133) |  |  |  |
| Constant | 2.471 |  |  |
| SFS (OSFS vs VSFS) | 0.440 | 0.180-1.076 | 0.072 |
| Social index (high vs low) | 3.539 | 1.225-10.224 | **0.020** |
| Social index (middle vs low) | 3.916 | 1.359-11.281 | 0.011 |
| χ²(3)=12.078, **p=0.007**, Nagelkerke’s *R*²=0.135 |  |  |  |
| **The menu is designed to suit the target group** |  |  |  |
| Constant | 1.493 |  |  |
| SFS (OSFS vs VSFS) | 0.169 | 0.076-0.371 | **<0.001** |
| Social index (high vs low) | 0.764 | 0.302-1.904 | 0.570 |
| Social index (middle vs low) | 0.632 | 0.251-1.594 | 0.331 |
| χ²(3)=23.211, **p<0.001**, Nagelkerke’s *R*²=0.201 |  |  |  |
| **Organic products are used** |  |  |  |
| Constant | 0.667 |  |  |
| SFS (OSFS vs VSFS) | 2157713066.045 | 0.000 | 0.996 |
| Social index (high vs low) | 0.675 | 0.205-2.222 | 0.518 |
| Social index (middle vs low) | 2.591 | 0.810-8.287 | 0.109 |
| χ²(3)=74.722, **p<0.001**, Nagelkerke’s R²=0.570 |  |  |  |
| **Fair trade products are used** |  |  |  |
| Constant | 0.057 |  |  |
| SFS (OSFS vs VSFS) | 0.845 | 0.254-2.811 | 0.783 |
| Social index (high vs low) | 1.599 | 0.277-9.224 | 0.600 |
| Social index (middle vs low) | 2.310 | 0.441-12.105 | 0.322 |
| χ²(3)=1.200, p=0.753, Nagelkerke’s *R*²=0.019 |  |  |  |
| **Sea fish from not overfished stocks** |  |  |  |
| Constant | 0.698 |  |  |
| SFS (OSFS vs VSFS) | 21.970 | 8.077-59.765 | **<0.001** |
| Social index (high vs low) | 0.258 | 0.084-0.792 | **0.018** |
| Social index (middle vs low) | 1.147 | 0.405-3.252 | 0.796 |
| χ²(3)=62.842, **p<0.001**, Nagelkerke’s *R*²=0.471 |  |  |  |
| **Meat from animal friendly husbandry** |  |  |  |
| Constant | 0.034 |  |  |
| SFS (OSFS vs VSFS) | 2.099 | 0.479-9.203 | 0.325 |
| Social index (high vs low) | 1.707 | 0.294-9.928 | 0.551 |
| Social index (middle vs low) | 0.740 | 0.099-5.521 | 0.769 |
| χ²(3)=1.917, p=0.590, Nagelkerke’s *R*²=0.038 |  |  |  |
